# Brain-CODE: A Secure Neuroinformatics Platform for Management, Federation, Sharing and Analysis of Multi-Dimensional Neuroscience Data

**DOI:** 10.3389/fninf.2018.00028

**Published:** 2018-05-23

**Authors:** Anthony L. Vaccarino, Moyez Dharsee, Stephen Strother, Don Aldridge, Stephen R. Arnott, Brendan Behan, Costas Dafnas, Fan Dong, Kenneth Edgecombe, Rachad El-Badrawi, Khaled El-Emam, Tom Gee, Susan G. Evans, Mojib Javadi, Francis Jeanson, Shannon Lefaivre, Kristen Lutz, F. Chris MacPhee, Jordan Mikkelsen, Tom Mikkelsen, Nicholas Mirotchnick, Tanya Schmah, Christa M. Studzinski, Donald T. Stuss, Elizabeth Theriault, Kenneth R. Evans

**Affiliations:** ^1^Ontario Brain Institute, Toronto, ON, Canada; ^2^Indoc Research, Toronto, ON, Canada; ^3^Rotman Research Institute, Toronto, ON, Canada; ^4^Centre for Advanced Computing, Kingston, ON, Canada; ^5^Privacy Analytics Inc., Ottawa, ON, Canada; ^6^Department of Mathematics and Statistics, University of Ottawa, Ottawa, ON, Canada; ^7^Departments of Psychology and Medicine, University of Toronto, Toronto, ON, Canada

**Keywords:** Brain-CODE, neuroinformatics, big data, electronic data capture, open data

## Abstract

Historically, research databases have existed in isolation with no practical avenue for sharing or pooling medical data into high dimensional datasets that can be efficiently compared across databases. To address this challenge, the Ontario Brain Institute’s “Brain-CODE” is a large-scale neuroinformatics platform designed to support the collection, storage, federation, sharing and analysis of different data types across several brain disorders, as a means to understand common underlying causes of brain dysfunction and develop novel approaches to treatment. By providing researchers access to aggregated datasets that they otherwise could not obtain independently, Brain-CODE incentivizes data sharing and collaboration and facilitates analyses both within and across disorders and across a wide array of data types, including clinical, neuroimaging and molecular. The Brain-CODE system architecture provides the technical capabilities to support (1) consolidated data management to securely capture, monitor and curate data, (2) privacy and security best-practices, and (3) interoperable and extensible systems that support harmonization, integration, and query across diverse data modalities and linkages to external data sources. Brain-CODE currently supports collaborative research networks focused on various brain conditions, including neurodevelopmental disorders, cerebral palsy, neurodegenerative diseases, epilepsy and mood disorders. These programs are generating large volumes of data that are integrated within Brain-CODE to support scientific inquiry and analytics across multiple brain disorders and modalities. By providing access to very large datasets on patients with different brain disorders and enabling linkages to provincial, national and international databases, Brain-CODE will help to generate new hypotheses about the biological bases of brain disorders, and ultimately promote new discoveries to improve patient care.

## Introduction

The principles of data sharing as a catalyst for scientific discovery are widely recognized by international organizations such as the National Institutes of Health (2003), Wellcome Trust (2010) and Canadian Institutes of Health Research (2013). Historically, however, research databases have existed in isolation with no practical avenue for sharing or pooling medical data into high dimensional “big” datasets that can be efficiently compared across databases. Databases have their own sets of data standards, software and processes, thus limiting their ability to synthesize and share data with one another. To address this challenge, the Ontario Brain Institute (OBI) created Brain-CODE – an extensible, neuroinformatics platform designed to support curation, sharing and analysis of different data types across several brain disorders^1^. Brain-CODE allows researchers to collaborate and work more efficiently to understand the biological basis of brain disorders.

**FIGURE 1:**
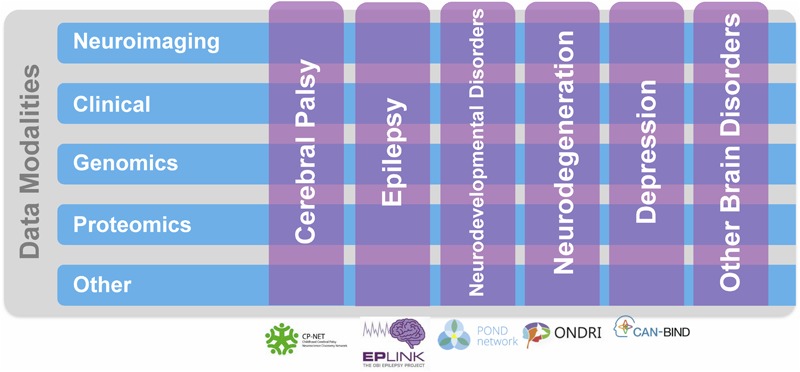
Ontario Brain Institute (OBI) Programs. These programs take a different approach to research that spans many disciplines and brings together a diverse group of stakeholders including researchers, clinicians, industry partners, and patients and their advocates. The programs collect various types of data, including genetic, molecular, imaging, and behavioral, which are stored on Brain-CODE. Figure adapted from Stuss (2014).

Ontario Brain Institute supports collaborative research networks focused on various brain conditions, including neurodevelopmental disorders^2^, cerebral palsy^3^, epilepsy^4^, mood disorders^5^, and neurodegenerative diseases^6^ (**Figure 1**). The creation of these programs has resulted in a “big data” opportunity to support the development of innovative, impactful diagnostics and treatments for brain disorders (Stuss, 2014, 2015). By providing researchers access to aggregated datasets that they otherwise could not obtain independently, Brain-CODE incentivizes data sharing and collaboration, and facilitates analyses both within and across disorders and across an array of data types, including clinical, neuroimaging, and molecular. By collecting data elements across disorders Brain-CODE enables deep phenotyping across data modalities within a brain disorder, as well as investigations across disorders. Moreover, linkages with provincial, national and international databases will allow scientists, clinicians, and industry to work together in powerful new ways to better understand common underlying causes of brain dysfunction and develop novel approaches to treatment.

Using the FAIR Data Principles as guidance, Brain-CODE is being developed to support the principles of data being Findable, Accessible, Interoperable, and Reusable (FAIR, Wilkinson et al., 2016). The Brain-CODE system architecture provides the technical capabilities to support:

• Consolidated data management to securely capture, monitor and curate data.
• Privacy and security best-practices.
• Interoperable and extensible federation systems that supports harmonization, integration and query across diverse data modalities and linkages to external data sources.

### Brain-CODE Design Principles

#### Interoperability and Standardization to Support Data Integration and Collaboration

The types of data being collected in modern research are increasingly diverse, from larger numbers of sources and patient populations, and involving highly specialized technologies, from genomics and imaging, to wearable devices and surveys delivered via mobile apps. There is also a growing need to link and query data collected within a given research study with data stored in disparate other locations and formats, such as public data repositories, health administration data holdings, electronic medical records, and legacy databases. As a result, researchers deploy a broad range of tools to collect, process and analyze their data, but the lack of interoperability of these platforms serves as a barrier to data sharing and collaboration. Establishing standard software would address these issues; however, available platforms each have their unique advantages and there is a significant cost for researchers in time and effort to move to new platforms. This complex set of data integration needs cannot be addressed using inflexible systems working in isolation nor by the development of a “one size fits all” platform. Rather, to support this level of data integration, interoperability must be a core requirement. This approach differs from most other data platforms in which data are combined at the data analyses stage. Interoperability, however, enables large-scale data aggregation and federation of systems and data across multiple data types, allowing novel discoveries and analyses to be conducted. Moreover, allowing researchers to decide which system to use ensures greater researcher uptake, which facilitates collaboration and data sharing within and across the broader research community.

From its inception, Brain-CODE architecture was designed with interoperability in mind, such that it could support the integration and analysis of large volumes of complex data from diverse sources. With this approach each platform can maintain its autonomy while still integrating into a much larger whole. This can be a challenge as databases are often stored in individual “silos” with their own sets of data standards, software and processes which limit their ability to interact with one another. Interoperability, therefore, requires the development of pipelines and processes between existing platforms; software to allow efficient and seamless exchange of data and information between systems and technologies, including application programming interfaces (APIs) to allow data flow between applications. In addition, rigorous standardization processes are required that govern how information is recorded and exchanged in order to define and format the vast array of clinical, neuroimaging and molecular data, and to optimize federation by ensuring that data in one system is understood by another. Effort must, therefore, be devoted to creating standards across studies, including common data elements (CDEs) (i.e., the same endpoints applied to multiple studies), common ontologies (i.e., utilizing common nomenclature and format across studies), as well as standard processes and procedures related to the collection of data.

#### An Extensible Design to Accommodate Expanded or Modified Functionality

Since not all functionality can be determined upfront, extensibility of the system must also be considered a core design principle to accommodate new and expanded functionality without impacting existing features. This approach allows the integration of users’ programs and third-party software into the system, as well as allowing for customization and enhancement of existing systems. Choice of technologies used and how the databases are built is critical and the use of commercial software can limit the ability to extend functionality, as these are typically built for a specific purpose and source codes are often not available. Extensibility is less of an issue when using open source software, as the code is published and can be modified. Where possible, therefore, Brain-CODE infrastructure is built using open-source tools.

#### Privacy and Security

Brain-CODE was designed with best-practice privacy strategies at the forefront to enable secure capture of sensitive participant data in a manner that abides by ethical principles and government legislation while fostering data sharing and linking opportunities. As such, privacy and security features have been robustly incorporated into the foundation of Brain-CODE’s infrastructure, and are reinforced by guidelines and safeguards that ensure participant data security.

Federation and linking with other databases involves the implementation of high-security data transfer infrastructure. These include encryption and de-identification tools to protect participant data and enhanced validation certificates to guarantee authenticity of outward-facing software applications, as well as administrative, physical and technical safeguards and security processes that are aligned with Code of Federal Regulations Title 21, Part 11 standards (CFR Title 21, Part 11, 2017). As a result, OBI has been named a “Privacy by Design” Ambassador by the Office of the Information and Privacy Commissioner of Ontario (Cavoukian, 2011). This designation refers to the mitigation of privacy and security risks through a proactive and preventative approach to research data management by embedding privacy and security measures directly into the design of systems and practices. Working with a team of experts, OBI has developed clear and comprehensive policies and guidelines on data privacy and governance^7^. These documents outline how data are collected, stored, and accessed by Brain-CODE users.

## Data Life-Cycle

**FIGURE 2:**
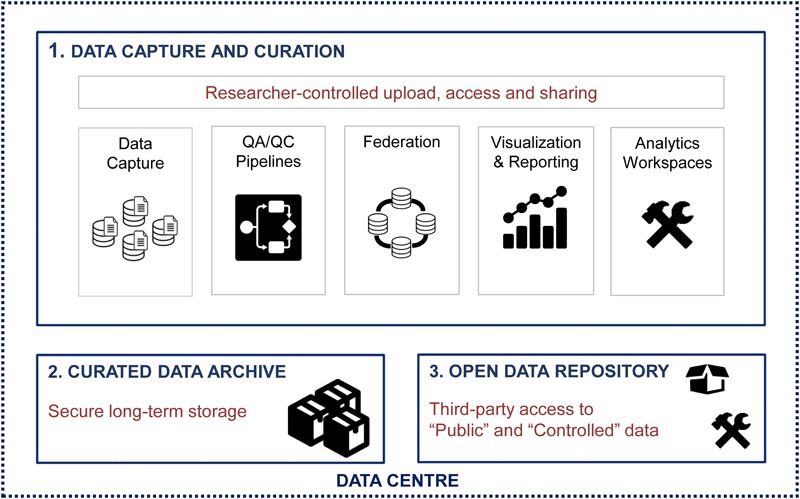
Data life cycle within Brain-CODE.

Researchers collect sensitive participant data in the form of clinical assessments, interventional studies, and brain imaging, cognitive and sensory-motor measures, as well as biological samples for proteomic and (epi-)genetic analyses. Personal Health Information (PHI) must be carefully handled in accordance with the Personal Health Information Protection Act, 2004, S.O. 2004, c. 3, Sched. A (Personal Health Information Protection Act [PHIPA], 2004) from a governance and contract perspective, as informed by principles in ISO 27001 for information management. To maximize the data sharing and analytics capacity of Brain-CODE, while enabling the secure collection of PHI, processes were developed to permit functional separation of sensitive data while being complemented by granular access controls to ensure that data are only available to Brain-CODE users who are authorized to access it (**Figure 2**).

### Data Capture and Curation

Brain-CODE provides a virtual laboratory environment where researchers (data producers) can upload, download, manage, curate and share their own research datasets with direct study collaborators. Based on Research Ethics Board (REB) approval and participant informed consent, data uploaded to Brain-CODE may include PHI. Before any data are uploaded to Brain-CODE, institutions enter into a Participation Agreement with OBI, whereby the institution and affiliated researchers agree to make use of the platform in a manner that abides by OBI’s Informatics Governance Policy, Platform Terms of Use and applicable privacy laws, and particularly institutional REBs. The participating institutions also grant OBI a non-exclusive license to share de-identified study datasets in the future, following an exclusivity period. An exclusivity plan is established between OBI and the researchers; during the period of exclusivity, data access remains exclusive to data producers and their direct collaborators. Before, during and after the exclusivity period, data producers and direct collaborators continue to have full access to their data, including access to a suite of analytical tools and workspaces, to enable data cleaning, curation and analysis required by studies.

### Curated Data Archive

Following an exclusivity period, curated datasets are versioned for long-term secure storage. These data are labeled as either “Controlled Data” or “Public Data.” Controlled Data are datasets that have been de-identified. These Controlled datasets are made available to third-party Brain-CODE users by request, and can be augmented through links to external databases in a secure environment. Public Data are either basic science datasets (i.e., from animal model studies), metadata, or human datasets that did not previously contain PHI. Public datasets can be shared directly with Brain-CODE users, without requiring an access request.

### Open Data Repository

**FIGURE 3:**
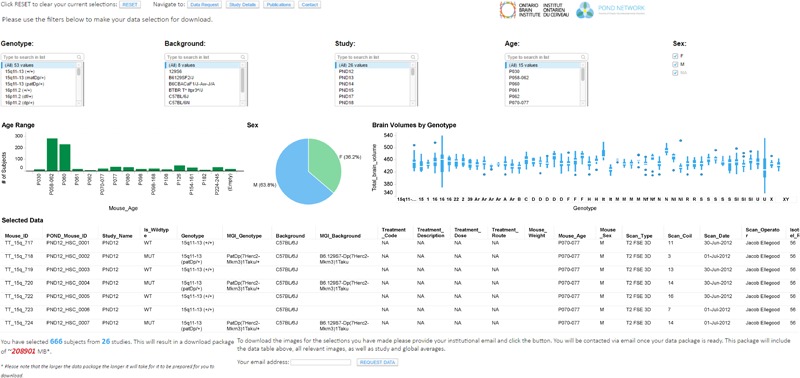
Data exploration and release dashboard. Open data release of High Resolution MRI of Mouse Models Related to Autism. Available at www.braincode.ca

One of the goals of Brain-CODE is to release high quality research data to researchers outside of OBI. The “Open Data” interface was developed for third-party users to browse information about Controlled and Public datasets and access data releases (see **Figures 2, 3**). While Public Data can be accessed directly, Controlled Data requires users to submit data access requests. Data access requests are reviewed by Brain-CODE’s Data Access Committee (DAC) which is composed of researchers, neuroinformatics experts, and OBI staff. The DAC makes a recommendation to the Informatics Steering Committee, which makes final decisions related to data access. Once a request is approved, third-party users must provide proof of REB approval and enter into a Data Use Agreement with OBI before being granted access to the data for retrieval and analyses. The de-identified dataset can be exported to a workspace environment available upon request to any registered Brain-CODE user, allowing access to high performance computing resources and analysis tools. The access request, review and approval process is streamlined within the Brain-CODE portal to ensure a timely turnaround of 10 days from access request to granting data access.

## Consolidated Data Management

**FIGURE 4:**
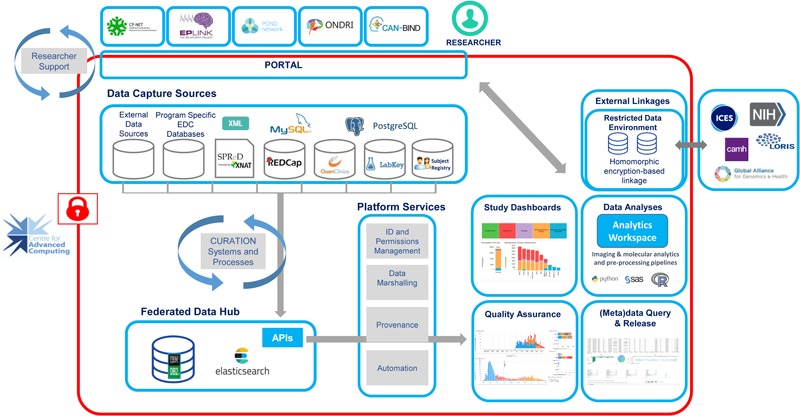
Brain-CODE system architecture.

Within the Brain-CODE Portal, data capture is consolidated with a diverse set of electronic data capture (EDC) tools for various data modalities including clinical, imaging, and ‘omics that allow researchers to securely upload, store and manage research data electronically^8^ (**Table 1**). The Brain-CODE platform was developed to allow incorporation of new data capture systems as required by the various research teams (**Figure 4**). In addition to providing a single point of access to data management tools, the Brain-CODE Portal features project management dashboards, private file repositories and discussion for a that researchers can use to facilitate sharing and collaboration.

**Table 1:** Data modalities currently collected in Brain-CODE.

Modality
Demographics
Patient-reported outcomes
Clinician-reported outcomes
Cognitive assessments
Structural MRI
Functional MRI
Diffusion tensor imaging (DTI)
Spectral MRI
Behavioral outcome files (timing, events)
Investigators notes
Electroencephalogram (EEG)
Electrocardiograph (ECG)
Pulse plethysmograph (PPG)
Respiratory
Magnetoencephalogram (MEG)
Ocular computed tomography (OCT)
Fundal photography
Eye tracking
Pupil metrics
Gait track data
Accelerometers
Force plate
Audio files
Video files
Pathology images
Imaging manual QC
fBIRN fMRI imaging metrics
OHIP numbers
Genotyping
ONDRISeq
SNP and expression arrays
GWAS
Sequencing (NGS)
Proteomics
Absorbance based assays (i.e., ELISA, etc.)

As with most data repositories, naming conventions standards are key. Not only do these features enable a given subject’s data to be linked across the corresponding data stored on separate data capture systems (e.g., that same subject’s clinical data stored on REDCap system with imaging stored on XNAT), but such standardization ensures that automated quality assurance (QA) and quality control (QC) pipelines can be successfully applied to the data. The naming format used across Brain-CODE programs conform to the general format of PPPTT_HHH_SSSS, where PPP is the program code, TT is the study code, HHH is the site code and SSSS is the subject number. The four-digit subject number is typically assigned by the subject co-coordinator. A fifth Subject ID digit can be employed if deemed necessary.

### Clinical Data Management

A core objective of Brain-CODE is to organize, standardize, and integrate the various forms of clinical information collected from OBI-funded and partner research programs. Traditionally, data have been collected on paper but there is a growing trend both in industry and academic research settings toward EDC for some forms of data (Food and Drug Administration [FDA], 2013). However, many academic research teams lack the necessary infrastructure and specialized skills to use and maintain a clinical data management system. To alleviate this situation, multiple web-based clinical data management software packages are deployed and hosted in Brain-CODE to allow researchers to remotely access these tools and integrate them into daily research practice.

The two primary clinical data management systems used to collect demographic and clinical data are REDCap (Research Electronic Data Capture^9^) and OpenClinica^10^. REDCap is a web-based application developed by a multi-institutional consortium led by Vanderbilt University specifically to support data capture for academic research studies. The software is freely available under the conditions of an end-user license agreement, and has been designed to be very simple to configure, use and maintain. As such, REDCap has grown into a very popular solution within the research community. REDCap is designed to comply with the United States’ Health Insurance Portability and Accountability Act of 1996 (HIPAA) regulations, but is currently not CFR Title 21, Part 11 compliant. OpenClinica is developed and maintained by OpenClinica LLC, in both an open source Community Edition as well as a commercially licensed Enterprise Edition, the latter providing training and technical support. The Enterprise Edition is currently deployed in Brain-CODE; both a development/test and a production instance are installed. OpenClinica LLC fully maintains the deployment, including installation validation, database backup configuration, OS updates, software patches and upgrades, and technical support. OpenClinica is a fully featured, web-based system that supports multi-site clinical trials and clinical data management. The software is compliant with HIPAA and CFR Title 21, Part 11, providing the required electronic signature and audit trail functionality for use in clinical trials requiring FDA regulatory approval. Additional clinical data capture systems can be deployed as required.

#### Clinical Data Standardization

Brain-CODE includes multidisciplinary collaborative research networks across multiple brain disorders. Given the different research aims, study designs and technologies used across research programs, establishment of a minimum set of clearly defined and standardized assessments across studies is essential to facilitate data sharing and integration, and to conduct meaningful analyses across disorders. Indeed, these data must be sufficiently comparable to allow any levels of data integration, and in the absence of common measures and data standards it is difficult to compare the results from one study to another. From a data integration perspective, CDEs and other standardized variables represent shared attributes between different data models that can significantly enhance the implementation of the federated database by reducing the semantic and syntactic heterogeneities between constituent databases. Therefore, in an effort to optimize the ability to aggregate and analyze data within Brain-CODE, CDEs were developed to provide standard definitions and formats so that investigators collect data consistently across studies and programs.

Using the framework of the National Institute of Neurological Disorders and Stroke (NINDS) CDE Project as guidance (Grinnon et al., 2012), a Delphi consensus-based methodology (Dalkey and Helmer, 1963; Hsu and Sandford, 2007) was used to identify core demographic and clinical variables to be collected across all participating OBI research programs. The CDEs include standardized assessments across the life-span of quality of life, medical and psychiatric co-morbidities, as well as clinical outcome measures of depression, anxiety, and sleep (**Table 2**). There was also agreement that when possible, the measure should be patient-reported, brief and easy to administer, widely used and validated, and available in the public domain. In addition, where possible the Clinical Data Interchange Standards Consortium (CDISC) standards are applied to define data collection fields, formatting, and terminology (Souza et al., 2007). This reduces variability in data collection and ultimately facilitates comparisons across disorders, merging of datasets and meta-analyses.

**Table 2:** Summary of Brain-CODE core demographic and clinical CDEs.

Domain	Sub-domain	Brain-CODE CDE
**Patient**	Demographic	Sex, DOB, Handedness, Ethnicity
**Characteristics**	SES	Education, Marital Status, Occupation, Income
**Physical and Mental Health**	Quality of Life	WHO Quality of Life -Short Version^1^ KINDL-R^2,3^
	Activities of Daily Living	Sheehan Disability Scale^1^
	Medical Comorbidity	NINDS Medical History^1,2,3^
	Psychiatric Comorbidity	Brief Symptom Inventory^1,2^
**Clinical Endpoints**	Depression	Quick Inventory of Depressive Symptomatology^1^ Revised Children’s Anxiety and Depression Scale^2,3^
	Anxiety	Generalized Anxiety Disorder-7^1^ Revised Children’s Anxiety and Depression Scale^2,3^
	Sleep	Pittsburgh Sleep Quality Index^1,2^ Children’s Sleep Habits Questionnaire^3^

*^*1*^Adult; ^*2*^adolescent; ^*3*^child.*

#### Clinical Data Quality Assurance and Control

Prior to data collection, clinical databases are validated to ensure adherence to data standards, compliance with the Brain-CODE CDEs, potential governance and privacy issues, and database quality. Identifying fields are compared against the language used in their ethics submission for compliance. Validation can also identify errors or missing data points in the database before data entry begins. Project validation involves a thorough review of a project’s variable naming, field naming, item coding, field validation and case report form equivalence through data entry, the data dictionary and data exports. This process is partially automated against a library of existing scales where possible. For novel forms, the digital version of the form is compared to the paper form and scoring manual as well.

Once collected, data cleaning and curation is typically supported within the clinical EDC system. REDCap users have the option to use REDCap’s API to extract data directly into a Brain-CODE workspace, allowing users to extract, subset and analyze their data, entirely within Brain-CODE’s secure environment. By extracting the data directly into a workspace, researchers avoid any errors potentially introduced by spreadsheet software, or through encoding conversion issues. For large collaborative studies having a centralized way for multiple users to run outlier analysis scripts in the same environment can help save data analysis resources required to reconfigure pipelines between different users’ institutional and personal computers. After the data are exported from the EDC system they will typically be manually reviewed against source documentation, or run through a curation pipeline to detect any outlying erroneous or aberrant data points. Those data points are then reviewed and if appropriate corrected in the source data, or noted as true outliers by the study teams in the data capture system itself alongside the raw data.

### Imaging Data Management

Many of the studies hosted on Brain-CODE collect various forms of medical imaging, with a particular focus on Magnetic Resonance Imaging (MRI). Although many different scanners are used across the various research sites, all the scanners provide data in the Digital Imaging and Communications in Medicine (DICOM) format^11^. The open source XNAT (eXtensible Neuroimaging Archive Toolkit) project by the Neuroinformatics Research Group at Washington University in St. Louis (Marcus et al., 2007) is used within Brain-CODE to gather, organize, query, and control access to MRI and related data. In addition to DICOM data, XNAT at Brain-CODE is also used to organize and assemble other large binary datasets, including magnetic encephalography (MEG), electrocardiography (ECG), electroencephalography (EEG), ocular computed tomography (SD-OCT), fundal photography, accelerometer and instrumented gait tracking data. Several forms of data that are required to interpret the scans are also included, such as output from stimulus presentation systems such as E-Prime^®^ and even simple scans of hand-written notes taken during sessions.

Data are uploaded via a secure web page into XNAT, either via manual transfer or through bulk upload via scripts. Using sophisticated DICOM interpretation capabilities, XNAT organizes the input files into appropriate sessions, which can be confirmed by the user or the upload script. Once in place, the system generates visual thumbnails, as well as populates a PostgreSQL database with metadata from the DICOM headers as well other environmental sources. These data are made available for searching, retrieval of metadata, and download, under a well-defined authorization structure.

#### Imaging Standards

The Brain-CODE’s XNAT file structure is hierarchically organized, with Project ID folders (i.e., PPPTT_HHH, see naming convention standards in section “Consolidated Data Management” above) occupying the highest level and containing the brain imaging data for that particular project. Within the Project ID folders are a series of subject folders (i.e., PPPTT_HHH_SSSS), each containing Session ID folders of brain imaging data from one or more distinct testing sessions from that subject. A Session ID always begins with a Subject ID, followed by an underscore and a_2-digit Visit ID, and then “_SE” and a 2-digit session number (i.e., ‘PPPTT_HHH_SSSS_02_SE01_MR’), as well as an optional “part code” which, if present, is a single lower case letter used to identify and link sessions that were broken up or spread out over time (e.g., intervening days as is the case with MR rescan requests). The session number is then followed by an underscore and a Modality code, which is a string of 2–4 characters indicating the imaging/recording modality.

Anyone requested by the program manager can be given read-only access to a Project folder, while only program manager-approved users who have also taken and passed an upload training tutorial on a non-production version of XNAT can be given upload access to such project(s). In order to reduce the chance of upload errors, XNAT uploaders are given the opportunity to review files and correct any issues at a pre-archive stage. Once archived, however, only Brain-CODE administration staff is allowed to amend files, and only at the written request of uploaders. To ensure provenance and prevent accidental data loss, data are not actually deleted, but session names have the suffix ‘_deleted’ added to them so that the files can be excluded from eventual curation. The one exception to this non-deletion rule pertains to uploaded data that violate ethical restrictions.

#### Imaging Quality Assurance and Control

Imaging data undergo multiple QA/QC steps as well as curation. Some forms have built-in support via XNAT, such as the manual QC reports, while others represent custom extensions to the basic system. Such custom extensions were started in the Stroke Patient Recovery Research Database (SPReD) (Gee et al., 2010) and extended within Brain-CODE so that the neuroimaging component is referred to as SPReD powered by XNAT, which we will simply refer to as XNAT. The extensive back-end API supported via a representational state transfer (REST) interface allows many manual and automated pipelines to be connected to XNAT, providing automated image transformation, conversion, evaluation and process coordination.

Multi-site brain-imaging studies offer many unique challenges compared to traditional single-site research approaches (see Farzan et al., 2017). Many of these become apparent when reviewing the QA and QC measures that are undertaken for imaging data on Brain-CODE. There are several QA and QC pipelines that are employed on Brain-CODE‘s XNAT. Due to its DICOM format, many of these pipelines cater to MR data.

##### SPReD/XNAT naming consistency QC

The naming consistency pipeline is a Python executable script that every night iterates through the data uploaded to SPReD/XNAT in Brain-CODE and checks whether the names of the uploaded files comply with the naming convention described above. In case a non-compliant name is found, the data uploader is notified by e-mail within 24 h, if the naming problem persists more than 7 days, the Program Manager is notified weekly by e-mail until the problem is corrected.

##### Scan acquisition protocol QC (pipeline operational for scanning sites)

**FIGURE 5:**
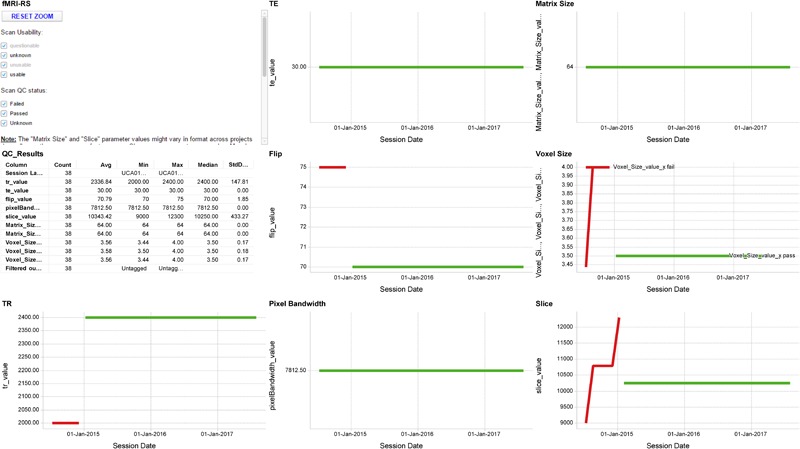
Sample MR QA Dashboard. Longitudinal display of parameter acquisition values obtained from one scanner’s monthly fMRI scans of an fBIRN phantom. Longitudinal results for the TR, TE, Flip angle, Pixel Bandwidth, Matrix size, Voxel size, and Slice Number parameters are displayed for 38 scans obtained between June 2014 and August 2017. Red and green traces indicate parameter values that deviate or fall within normal limits of the expected values, respectively.

The scan acquisition QC pipeline compares the parameters for all scans within an MRI session from a particular scanning site against a reference protocol defined by the relevant program. The protocols are configured on a project-by-project and scanner-by-scanner basis for each scanning site. The protocol defines a set of pulse sequences that should exist within the session, along with a set of values for the acquisition parameters for each sequence. Each parameter has an upper and lower value against which the actual scan parameters are evaluated. Within 24 h of a failure occurring for any parameter the Program and Brain-CODE neuroimaging managers are notified by e-mail and s/he will contact and work with the scanning site to try to ascertain and correct the cause of the failure. Protocol adherence is aggregated and displayed (**Figure 5**).

##### Manual/visual QC

It is strongly recommended to every program that they institute a manual visual inspection of all data uploaded to SPReD. The criteria for assessment is based on the Qualitative Quality Control Manual by Massachusetts General Hospital (2013). Results of the manual QC check are recorded in SPReD/XNAT and may be viewed and retrieved from the records of each scan session. If any acquisition fails manual QC the results are discussed with the scanning site within 48 h of the initial patient scan.

##### fMRI QA pipeline for fBIRN phantom

The goal of the fBIRN phantom and pipeline software from the Biomedical Informatics Research Network is to provide QA tools for tracking functional MRI (fMRI) imaging performance (Friedman and Glover, 2006). OBI scanning sites have an fBIRN phantom purchased for them by OBI. These phantoms are scanned on a monthly basis and uploaded to XNAT. The fBIRN QA pipeline is then automatically run on these data within 24 h of upload, and a full QA report is generated and stored within the session. The phantom and QA procedures are more formally described in Friedman and Glover (2006), and Glover et al. (2012). Tools for tracking these QA results over time and notification thresholds for scanning sites have been developed using dashboards visualizations. Currently a site is notified if any derived phantom parameter differs from its mean by more than 3 Standard Deviations, based on all previous values acquired to date.

##### DTI QA pipeline for fBIRN phantom

The utility of the fBIRN spherical gel phantom has been extended to monitoring the performance of DTI acquisitions (Chavez et al., 2018). As is the case for the fMRI QA results, tools for tracking these DTI QA results over time and notification thresholds for scanning sites are available as dashboards.

##### fBIRN fMRI human QC pipeline

A goal of the Biomedical Informatics Research Network is to provide QC tools for tracking functional MRI imaging performance. A full QC report (index.html) for every fMRI scan generated by running the fBIRN phantom and the fBIRN human pipeline software packages on human data is available through the Brain-CODE XNAT file manager in the scan’s session folder. Tools for tracking these QC results over time and notification thresholds for scanning sites are available as dashboards.

##### LEGO phantom QA/QC pipeline

The LEGO phantom and associated pipeline are designed to measure and correct for magnetic field gradient induced geometric distortion, and thereby reduce measurement variability of morphometric measurements from high-resolution T1 MRI scans. The pipeline procedure and its impact on morphometric measurements in neurodegeneraton are described in Caramanos et al. (2010).

##### MRI registration QC pipeline

The MRI registration pipeline automatically registers (non-linear warping with ANTS^12^) every new high-resolution T1 MRI structural scan to a template and then automatically measures signal-to-noise (SNR) and contrast-to-noise (CNR) in gray matter. The pipeline also includes white matter and automatically measured volumes of interest using the MNI152 registration template and the LPBA40 segmentation atlas (Shattuck et al., 2008).

##### DICOM header de-identification pipeline

Brain-CODE also employs a number of security pipelines for imaging data. The de-identification pipeline is configured to remove or replace a set of fields within the header of MRI DICOM files and employs a fixed set of fields to be cleared or modified. The appropriate set of fields needs to be reviewed by the users, as they may vary somewhat between projects, between scanners and even between scanner software revision levels.

##### Defacer pipeline

The Deface DICOM pipeline removes facial features from a DICOM-format T1 image, and produces a defaced DICOM image that is identical to the original in all other respects. It is based on the mri_deface tool released with FreeSurfer and described in Bischoff-Grethe et al. (2007). The output of mri_deface is in Neuroimaging Informatics Technology Initiative (NIfTI) format. The pipeline converts this to DICOM, using the original DICOM file set and the tools mri_convert and analyze2dcm.

##### Virus pipeline

All new files in the SPReD/XNAT database are scanned for viruses every 24 h.

### ‘Omics and Molecular Data Management

Many of the participating studies collect various molecular and ‘omics data as biomarkers for diagnosis and prognosis of disease (Lam et al., 2016; Farhan et al., 2017). Ultimately, Brain-CODE federates these various molecular data modalities with the clinical and imaging data also being collected in these studies, enabling integrated query and analysis of these complex datasets. Brain-CODE currently utilizes the LabKey Server Community Edition, an open source web server developed by the LabKey Corporation^13^. LabKey provides an array of features crucial in efficient management and organization of molecular data from sample tracking, to file archiving to tabularization of finalized datasets. LabKey provides both technology/assay-specific as well as customizable data schemas, making it a flexible and scalable solution for dealing with the large variety of data types being collected by the Brain-CODE-supported studies. Additionally, LabKey provides a suite of intuitive collaboration features, making it more efficient for investigators across multiple sites to coordinate biological samples, processing and analysis of data.

The installation of LabKey within Brain-CODE provides researchers from multiple labs with a centralized location for the collection and tracking of sample information, raw data files, processed data and associated metadata, including protocol and experimental details, QA/QC and processing metadata of samples and resulting data. Projects are set up to ensure that all these components are appropriately integrated, making it easy to obtain query-based data cuts of processed data and raw data files. Additionally, where possible, final processed data points are structured into a Postgres database which enables more granular and in-depth integrated queries of the molecular datasets with other data modalities. This provides a challenge as ‘omics datasets expand in size and complexity, requiring scalable query solutions that can be integrated into existing systems.

#### ‘Omics and Molecular Standards

Centralized management of ‘omics and molecular data introduce a unique set of challenges including a very diverse set of data modalities, large and ever-growing datasets and files, and harmonization with prominent ‘omics databases, like the Gene Expression Omnibus (GEO), GenBank, Sequence Reach Archives, and existing standards [i.e., Minimum Information About a Microarray Experiment (MIAME), Minimum Information about a high-throughput nucleotide SEQuencing Experiment (MINSEQE), Global Alliance for Genomics and Health (GA4GH) and others]. Brain-CODE takes advantage of existing standards and workflows to ensure a thorough capture of all data and associated metadata, while harmonizing with the upload processes of prominent ‘omics databases. This in turn makes future submission of data prospectively collected on Brain-CODE simpler for the data producer.

### Data Query and Visualization

Several levels of query access are possible on the Brain-CODE system (see **Figure 4**). At the project level, researchers may query their own data within the applicable Brain-CODE data collection platform(s). Post-federation, the Brain-CODE data warehouse structure allows for flexibility in query methodology using either traditional relational database approaches (Structured Query Language, SQL) or unstructured methods such as Lucene via ElasticSearch^14^. This approach allows for future scalability as additional studies and data collection platforms are added to the Brain-CODE system. Metadata compiled for each study are stored in the Brain-CODE system and provides additional context to the data tables.

At the federation level, raw and/or curated federated datasets appropriate to the stage of the Brain-CODE data life-cycle are compiled for exposure to end users. Data visualization and query tools such as TIBCO Spotfire^15^ are employed to display and permit query of aggregated datasets across platforms and, if appropriate, permit users to access and download data tables. Alternative query tools can also be implemented to accommodate different data modalities. Security is ensured by means of user-based access controls at all levels of the data query system.

**FIGURE 6:**
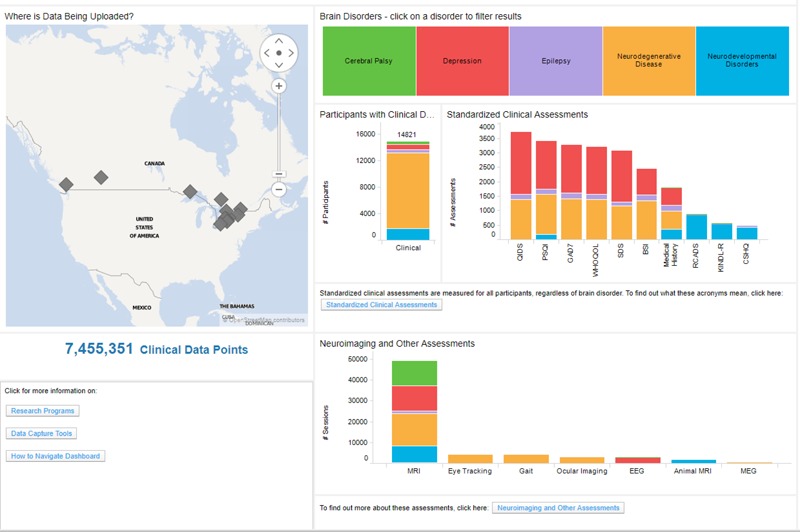
Data exploration dashboard showing summary of the data in Brain-CODE. Updated summary found at www.braincode.ca

Brain-CODE currently utilizes Spotfire to develop comprehensive administrative and analytic dashboards, providing unified views on integrated datasets stored in the platform’s federation system (**Figure 6**). This takes advantage of the continuous data federation across multiple data sources, allowing near real-time interaction with cross-project, multi-modal datasets. Administrative dashboards allow the Brain-CODE team to monitor the status of all studies on the Brain-CODE platform, describe and quantify data table properties, and apply global QC methods to ensure data quality across all studies and platforms. Project dashboards are configured to provide researchers with fully customizable views of the status of their studies (e.g., recruitment rates, participant profiles), ongoing QC and edit checks (e.g., missing data, protocol violations), and the ability to track ethics and informed consent restrictions. Data exploration and query dashboard interfaces enable permission-based sharing of data, both within study teams and with collaborators, and the broader research community.

### Analytics Workspace

Research groups utilizing Brain-CODE present highly variable computational needs during the data curation and analysis stages of their studies. Some are self-sufficient in their capacity to process large volumes of raw data such as MR images or DNA sequences, or to apply machine learning tools on high-dimensional datasets. For example, core sequencing labs used by some research groups have access to their own bioinformatics pipelines, server clusters and expertise required to conduct whole-genome variant detection, differential RNA quantification, or other analysis. Other groups are less equipped, wish to supplement their resources, or prefer to avoid the cost and risk associated with the transfer of large datasets and choose instead to carry out their computations where the data are already aggregated.

To this end, researchers can access a Brain-CODE analytics workspace, a secure environment with dedicated computing resources and necessary software to allow for specialized data processing and analyses. The term “workspace” is used broadly. It can be a cluster of Linux virtual machines (VMs) running the Slurm job scheduler for batch processing; a single Windows VM with SAS or SPSS installed; or an RStudio shared project accessed by data scientists from multiple locations. The analytics workspaces ensure the data are kept securely within the platform to satisfy any privacy and REB requirements while providing easy access to both the data and required resources.

### Subject Registry

When researchers enter or upload a dataset for a given participant, a standard Brain-CODE subject ID is assigned. A unique index of projects and Subject IDs is maintained in the Brain-CODE “Subject Registry,” which regularly collects all subject identifiers from the domain-specific databases and provides QC functionality. This critical integration between each database system and the Subject Registry is implemented through a “Reporter” application which extracts necessary information from the database (e.g., the Subject ID) and reports the information to the Subject Registry over REST-based web services.

The Subject Registry also provides functionality for encryption of PHI that can be used to link participant level information across databases, such as a health plan or medical record numbers. Encryption is performed within the user’s web browser, and the original value of the element never leaves the research site; only the ciphertext is transmitted and stored in the Subject Registry. Furthermore, the private key required for decryption is maintained by a third-party and is not known to Brain-CODE. The encryption algorithm has a particular homomorphic property which allows mathematical operations and comparisons to be applied to the encrypted data itself, i.e., without the need for decryption. These encryption capabilities not only provide robust safeguards against re-identification of sensitive data, they also enable secure data integration. For example, using a common identifier such as the Ontario Health Insurance Plan number, research data stored in Brain-CODE can be securely linked with administrative health databases such as the Institute for Clinical and Evaluative Sciences without requiring either party to disclose PHI.

### Privacy and Security

#### PHI and De-Identification

To protect the privacy and confidentiality of individuals and security of data held in Brain-CODE, OBI has adopted a Privacy-by-Design approach to creating and implementing protective measures. This policy is specific to Brain-CODE and is based on the 10 Canadian Standards Association (CSA) Privacy Principles (Canadian Standards Association [CSA], 1996). To ensure that privacy is not compromised, direct identifiers that provide an explicit link to a study participant and can identify an individual (i.e., health card number) are removed (or encrypted) to the extent possible. Nonetheless, Brain-CODE may include personal health information that has been collected for the purposes of the research study and analyses (i.e., date of birth). When such information is required and informed consent has been obtained, only researchers involved in the study will have access to it in a firewalled and secure environment. Prior to disclosure to third parties, direct identifiers are removed (or encrypted) to the extent possible.

#### Ethics Tracking and Monitoring

Brain-CODE operates based upon informed participant consent, meaning that institutional REB approvals and associated informed consents govern which data can be collected, uploaded, de-identified, and shared on Brain-CODE. This information is tracked in a centralized Brain-CODE Ethics Tracking Database, which contains information on the sensitivity of datasets and sharing permissions. The information in the Ethics Tracking Database is linked to each participant via the Subject Registry which allows the tracking and management of data permissions on a participant-by-participant basis.

## Data Federation and Linking

By design, research data stored in Brain-CODE are distributed over multiple distinct database applications, each with a unique underlying data model geared toward the capture of a subset of data modalities. There may be multiple systems in place to support a given modality. For example, clinical assessment data are captured in OpenClinica for some studies, and in REDCap for others. The choice of a clinical data management system for a given study is left to individuals involved in the study and to Brain-CODE personnel providing study support, who collectively take into consideration various factors such as regulatory requirements, training implications, specific features, etc. The same reasoning applies to data capture for other modalities, such as neuroimaging or molecular. While this approach provides maximum flexibility to researchers, allows use of best-of-breed systems developed by domain experts, and enables the platform as a whole to adapt and evolve according to changing needs, it does entail the technical challenge of systematic aggregation of data stored amongst several heterogeneous systems.

To make it possible to search, query, and extract these distributed data, Brain-CODE employs a hybrid “federated data hub” model whereby relevant data from each data source are harmonized and aggregated into one or more repositories (see **Figure 4**). APIs allow cross-system, and hence cross-modality query of federated data for diverse purposes by downstream systems, such as curation pipelines, interactive dashboards, search interfaces, and linkages with data systems external to Brain-CODE.

In its current implementation, federated data sources include: OpenClinica Enterprise^TM^; REDCap^TM^; Medidata RAVE^TM^; LimeSurvey^TM^; Subject Registry; XNAT; LabKey^TM^; LORIS (Das et al., 2012). The federated repository is implemented with a combination of IBM InfoSphere Federation Server^16^, which provides functions for extracting and staging source data into a DB2 relational database system, and Elasticsearch^17^, which provides functions to store data without the need of a pre-defined data model, and functions to index these data for very rapid searching. Query APIs consist of database-level functions and REST-based web services. Automated pipelines are implemented to extract data from source systems and ingest them into the repository. These pipelines execute at varying frequencies for different data types, depending on downstream data consumption needs; generally, federated data are refreshed daily.

Data records stored in the federated repository are associated with metadata. For participant records, these metadata include identifiers which point to the research project, data collection site, and participant associated with the data. Additional participant-related metadata include data sharing permissions derived from informed consent forms and institutional ethics review. These metadata provide a basis for access control implemented in downstream systems. This allows permission-controlled access by researchers to the data they collect from their own studies, as well as data collected across research programs. By federating data from multiple sources and data types, Brain-CODE provides researchers with unprecedented tools for combining, accessing and analyzing data in novel and powerful ways.

### Linkages With External Databases

To augment and complement data in Brain-CODE for enriched analysis and enhanced data outcomes, the system is also used to support linkages with data holdings external to Brain-CODE, such as public data repositories, health administration data holdings, electronic medical records, and legacy databases (see **Figure 4**). For example, a federation of clinical and neuroimaging data has recently been implemented between the Brain-CODE and the LORIS database hosted at McGill University (Das et al., 2012), initially to support data exchange between the OBI-funded Ontario Neurodegeneration Disease Research Initiative program^18^ and the Canadian Consortium on Neurodegeneration in Aging^19^. The aim of this project is to ensure that researchers using both platforms can exchange data in an interoperable fashion, with minimal interference to their workflows. This has also laid the groundwork for a recently funded Brain Canada Platform Support Grant, the Canadian Open Neuroscience Platform (CONP), designed to bring together existing Canadian neuroscience platforms, initiatives and networks, and allow them to link, leverage, enhance and expand to form an integrated network. Both LORIS and Brain-CODE platforms will be actively involved in the creation of the CONP. In addition, the system is being extended to enable linkages with other partners, including linking of single-subject data with administrative health data holdings at the Institute for Clinical Evaluative Sciences (Institute for Clinical and Evaluative Services [ICES], 2017), and at the cohort-level with the National Institute of Mental Health Data Archive (Ontario Brain Institute [OBI], 2015).

### Other Brain-CODE Deployments

Where possible, Brain-CODE infrastructure was built using open-source tools, which lends itself to replication at other institutions. As discussed elsewhere in this special issue (Rotenberg et al., in review)^20^, the Brain-CODE infrastructure has been installed as the central informatics platform for servicing the Krembil Centre for Neuroinformatics at the Centre for Addiction and Mental Health (CAMH). With common software packages installed and similar standardization procedures in place, the groundwork has been laid for other institutions to benefit from this integrative data analytics approach.

## Data Center

The computational infrastructure for Brain-CODE is provided and maintained by the Centre for Advanced Computing (CAC) at Queen’s University, in Kingston, Canada^21^. CAC is a member of the regional Compute Ontario consortium, and affiliated with the Compute Canada national network. The CAC currently supports over 800 research teams across Canada, including academic and industry organizations. Reliable high-speed connectivity with major computing and academic centers is enabled regionally by redundant CAC links to the Ontario Research and Innovation Optical Network (ORION) private fiber optic network, and nationally and internationally through the CANARIE high-speed national backbone. Security best practices including administrative, technical and physical safeguards, and rigorous enforcement of information security policies and procedures, ensure that the platform can satisfy the most stringent regulatory requirements pertaining to the storage and use of sensitive data.

The Brain-CODE deployment at CAC provides a robust, scalable, high performance computing platform that can satisfy long-term processing and storage requirements of multiple large scale research programs, while enabling secure and seamless open access data sharing and analysis, which includes a combined processing performance of 5 TFLOPS (Gee et al., 2010). As usage and requirements of Brain-CODE grow, additional hardware resources can be allocated for increased data storage, specialized data processing, added demand for federation, and intensive concurrent analytical tasks. Brain-CODE public-facing applications and internal systems, including databases, pipelines, and various data handling services, are all deployed with containerization and virtualization technologies (e.g., Docker), allowing optimal use of processor and memory resources while streamlining system maintenance, and enabling the platform to be readily scaled or redeployed into new environments.

## Discussion

Ontario Brain Institute supports multidisciplinary collaborative research networks from across Canada focusing on various brain conditions. These programs generate large volumes of data that are integrated within Brain-CODE to support scientific inquiry and analytics across multiple brain disorders and modalities, including clinical, imaging, and ‘omics data. By providing access to very large datasets on patients with different neurological disorders and enabling linkages to provincial, national and international databases, Brain-CODE will generate new hypotheses about brain disorders and underlying causes, and ultimately promote new discoveries to improve patient care. As of March 18, 2018, Brain-CODE supports the acquisition, storage and analysis of multi-dimensional data from over 40 Canadian institutions, supporting more than 600 users in over 100 studies and contains data from more than 17,000 study participants and 1,500 animal subjects^22^ (see **Figure 6**). These research programs are continually adding data and new programs are being added.

In addition to OBI-supported programs, Brain-CODE also supports the collection, storage and sharing of data from other studies as well. Depending on the requirements of the programs, these data can be collected within the current instance of Brain-CODE with appropriate access control provided to the researchers. Alternately, a Brain-CODE instance can be located within separate servers at the CAC or installed within a separate data center altogether, as is the case with the CAMH instance of Brain-CODE. To facilitate sharing of these data with OBI-sponsored programs, all studies are encouraged to incorporate Brain-CODE CDEs into their protocols, which are made publically available on the Brain-CODE portal^23^. Furthermore, as many granting agencies and journals now require that research data be available for re-use by others, Brain-CODE also provides the infrastructure to support the upload and sharing of data collected outside of Brain-CODE, which can be made publically available or with restricted access to specified persons. Although Brain-CODE does not currently support “regulatory-compliant” clinical trials, plans are well underway to ensure that both the infrastructure and processes are in place to support regulatory-complaint clinical trials, including support of 21-CRF Part 11 compliant EDC systems (i.e., OpenClinica Enterprise) and development and adherence to Standard Operating Procedures, which have been adopted from N2 Network of Networks^24^.

One of the key goals of OBI is to support a collaborative approach to neuroscience as a mechanism to bring researchers together to maximize their collective impact (Stuss, 2015; Stuss et al., 2015). To help track the impact of OBI-supported initiatives in fostering collaborations among Ontario’s neuroscience community, an “Atlas of Ontario Neuroscience” was developed to explore the growing collaborations both at the individual and institutional level^25^. For example, the “People Connection Map” shows collaborations OBI has fostered through Brain-CODE and other OBI-supported initiatives. It is expected that Brain-CODE, as a centralized informatics platform that supports the management, federation, sharing and analysis of multidimensional neuroscience data, will continue to strengthen and expand these collaborations not only within Ontario but also across the international neuroscience community.

## Author Contributions

All authors contributed to the development of Brain-CODE and commented on/revised the manuscript at all stages. AV wrote the first draft of the paper and prepared the manuscript.

## Conflict of Interest Statement

AV, MD, SA, RE-B, TG, SE, MJ, KL, JM, and KRE were employed by Indoc Research. KE-E was employed by Privacy Analytics, Inc. The other authors declare that the research was conducted in the absence of any commercial or financial relationships that could be construed as a potential conflict of interest.

## Footnotes

1 www.braincode.ca

2 www.pond-network.ca

3 cpnet.canchild.ca

4 www.eplink.ca

5 www.canbind.ca

6 www.ondri.ca

7 www.braincode.ca/content/governance

8 www.braincode.ca

9 projectredcap.org

10 www.openclinica.com

11 www.dicomstandard.org

12 http://sourceforge.net/projects/advants/

13 www.labkey.com

14 www.elastic.co

15 spotfire.tibco.com

16 www.ibm.com/analytics/information-server

17 www.elastic.co

18 www.ondri.ca

19 ccna-ccnv.ca

20 Rotenberg, D., Chang, Q., Potapova, N., Wang, A., Hon, M., Sanches, M., et al., The CAMH Neuroinformatics Platform: a hospital-focused Brain-CODE implementation. Submitted to Frontiers in Neuroinformatics.

21 https://cac.queensu.ca

22 www.braincode.ca

23 https://www.braincode.ca/content/getting-started

24 http://n2canada.ca

25 http://axon.braininstitute.ca/index.html
